# Asymptomatic primary tuberculous pleurisy with intense 18-fluorodeoxyglucose uptake mimicking malignant mesothelioma

**DOI:** 10.1186/1471-2334-13-12

**Published:** 2013-01-14

**Authors:** Tsutomu Shinohara, Naoki Shiota, Motohiko Kume, Norihiko Hamada, Keishi Naruse, Fumitaka Ogushi

**Affiliations:** 1 Department of Clinical Investigation, National Hospital Organization National Kochi Hospital, 1-2-25 Asakuranishimachi, Kochi, 780-8077, Japan; 2 Department of Hematology and Respiratory Medicine, Kochi Medical School, Kochi University, Nankoku, Kochi, 783-8505, Japan; 3 Department of Surgery II, Kochi Medical School, Kochi University, Nankoku, Kochi, 783-8505, Japan; 4 Department of Radiology, Kochi Medical School, Kochi University, Nankoku, Kochi, 783-8505, Japan; 5 Department of Pathology, Kochi National Hospital Organization National Kochi Hospital, 1-2-25 Asakuranishimachi, Kochi, 780-8077, Japan; 6 Division of Pulmonary Medicine, National Hospital Organization National Kochi Hospital, 1-2-25 Asakuranishimachi, Kochi, 780-8077, Japan

**Keywords:** Primary tuberculous pleurisy, Fluorodeoxyglucose, Positron emission tomography

## Abstract

**Background:**

The pathogenesis of primary tuberculous pleurisy is a delayed-type hypersensitivity immunogenic reaction to a few mycobacterial antigens entering the pleural space rather than direct tissue destruction by mycobacterial proliferation. Although it has been shown that pulmonary tuberculosis induces 18-fluorodeoxyglucose (FDG) uptake in active lesions, little is known about the application of FDG positron emission/computed tomography (FDG PET/CT) to the management of primary tuberculous pleurisy.

**Case presentation:**

We report a case of asymptomatic primary tuberculous pleurisy presenting with diffuse nodular pleural thickening without distinct pleural effusion and parenchymal lung lesions mimicking malignant mesothelioma. An initial FDG PET/CT scan demonstrated multiple lesions of intense FDG uptake in the right pleura and thoracoscopic biopsy of pleural tissue revealed caseous granulomatous inflammation. The patient received antituberculous therapy for 6 months, with clearly decreased positive signals on a repeated FDG PET/CT scan.

**Conclusion:**

FDG PET/CT imaging may be useful for evaluating disease activity in tuberculous pleurisy patients with an unknown time of onset.

## Background

18-fluorodeoxyglucose positron emission/computed tomography (FDG PET/CT) is a noninvasive imaging technique that visualizes glucose metabolism and has been regarded as useful for differentiating between benign and malignant diseases [1,2]. However, it has been shown that inflammatory disorders, especially pulmonary tuberculosis, also induce FDG uptake in active lesions, which suggests that FDG PET results should be interpreted with caution [3-5]. Conventional or dual phase FDG PET can not effectively distinguish malignant disease from mycobacterium infections. Multi-tracer PET, a novel promising technology utilizing differences in tracer kinetics and decay, has been providing additional and complementary information to improve pulmonary nodule characterization [5].

The usefulness of FDG PET in the detection of mycobacterium infection sites has been shown in patients with miscellaneous infectious lesions or human immunodeficiency virus-associated fever of unknown origin [6,7]. Moreover, it has been reported that FDG PET/CT is helpful not only for evaluating disease activity, but also for monitoring therapeutic responses in pulmonary tuberculosis, tuberculous lymphadenitis, and skeletal tuberculosis, particularly in smear-negative patients [5,8-10]. Via et al. demonstrated that FDG-PET uptake in tuberculous pulmonary lesions was significantly reduced with as little as 1 week of treatment prior to changes in the volume and density of lesions in a rabbit model [11]. The absence of reduced FDG PET uptake in the early phase of treatment may suggest active tuberculosis due to a lack of adherence, drug resistance, or misdiagnosis [9].

The pathogenesis of primary tuberculous pleurisy is a delayed-type hypersensitivity immunogenic reaction to a few mycobacterial antigens entering the pleural space rather than direct tissue destruction by mycobacterial proliferation [12]. However, little is known about the application of FDG PET/CT imaging to the management of primary tuberculous pleurisy. We report here a case of asymptomatic primary tuberculous pleurisy with intense FDG uptake mimicking malignant mesothelioma.

## Case presentation

**Figure 1:**
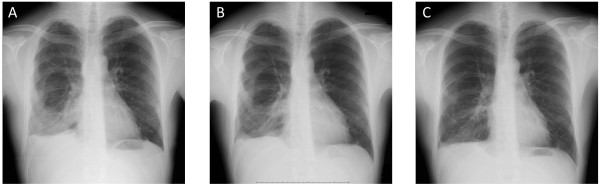
**Chest radiograph. A**: Pleural based opacities in the right hemithorax at presentation. **B**: Marked improvements after 6 months of antituberculous chemotherapy. **C**: Further improvements another 6 months after the completion of chemotherapy.

**Figure 2:**
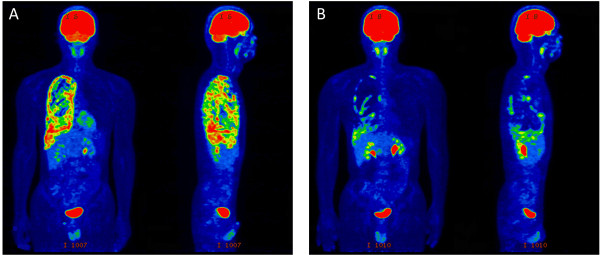
**FDG PET image. A**: Multiple lesions of intense FDG uptake in the right pleura at presentation. **B**: Clearly decreased positive signals after 6 months of antituberculous chemotherapy.

**Figure 3:**
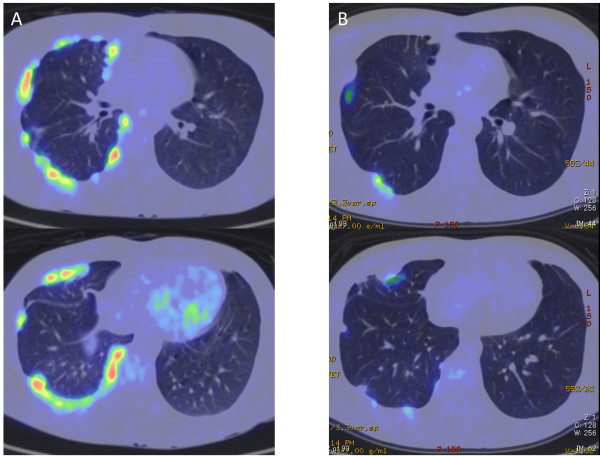
**FDG PET/CT scan. A**: Extensive nodular thickening of the right pleura at presentation showing intense FDG uptake without distinct pleural effusion and parenchymal lung lesions. **B**: Marked improvements with decreased positive signals after 6 months of antituberculous chemotherapy.

A 26-year-old man with no subjective symptoms was referred to our hospital because of a chest radiographic abnormality in the right hemithorax on his chest X-ray (Figure 1A), which was incidentally found during a college health examination. Chest CT revealed diffuse nodular thickening of the right pleura, without distinct pleural effusion and parenchymal lung lesions, mimicking malignant mesothelioma. An initial FDG PET/CT scan demonstrated multiple lesions of intense FDG uptake in the right pleura (maximal standard uptake value (SUVmax) = 10.9) (Figure 2A and Figure 3A). He had no history of pulmonary or autoimmune disease or occupational exposure to asbestos, silica, or other fibrogenic substances. Since the pleural specimens obtained from CT-guided needle biopsy were insufficient to allow for a definitive diagnosis, the patient was admitted for thoracoscopic biopsy.

**Figure 4:**
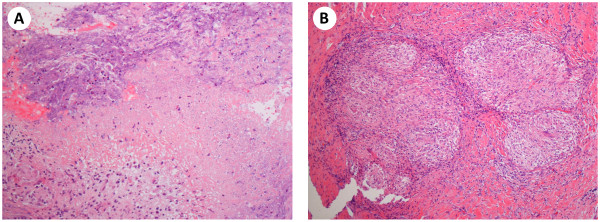
**Pathological findings of pleural tissue. A**: Caseous necrosis. **B**: Granulomatous inflammation.

On admission, right breath sounds were decreased. Remaining physical examinations revealed no abnormal findings. A full blood cell count, the results of liver and renal function tests, and other data were within normal limits. Thoracoscopic biopsy of the pleural tissue revealed caseous necrosis and granulomatous inflammation (Figure 4), although acid-fast bacilli were not found in biopsy specimens. Cultures of sputum and pleural tissue were also negative. However, the QuantiFERON-TB second generation (QFT-2G) test (3.02 IU/ml) and tuberculin skin test (10 mm induration) were both positive based on the Japanese criteria.

Although no positive culture was obtained, other clinical data suggested that a diagnosis of tuberculous pleurisy was highly likely in this case and the patient received antituberculous therapy for 6 months (2HREZ/4HRE) without any side effects. Just after chemotherapy, a repeated FDG PET/CT scan revealed clearly decreased positive signals (SUVmax = 5.7) with marked improvements in pleural thickening (Figure 2B and Figure 3B). Six months after the completion of chemotherapy, a chest radiograph showed further improvements in pleural based opacities relative to that taken just after chemotherapy (Figure 1B and C).

## Discussion

Pleural inflammation caused by bacteria, mycobacterium, or autoimmune disease is usually characterized by effusion, fever, malaise, chest pain, nonproductive cough, and dyspnea in the acute phage [13], or diffuse thickening of the pleura, with or without calcification, in the chronic phase. Therefore, pleural thickening with no subjective symptoms generally implies non-active inflammatory pleural diseases, so-called “residual pleural thickness”, or neoplasm of the pleura. Interestingly, the asymptomatic diffuse pleural thickening in our case showed intense FDG uptake and proved to be caseous granulomatous inflammation.

Duysinx et al. suggested that FDG PET/CT is an effective tool for differentiating between benign and malignant pleural diseases. The uptake of FDG was intense in all cases of mesothelioma and neoplastic pleurisy associated with primary lung cancer. In contrast, FDG PET/CT imaging revealed an absence of FDG uptake within the pleura of 2 of 3 patients with tuberculous pleurisy and the rest showed moderate uptake, although detailed clinical data of these patients were not described [14]. Kramer et al. also evaluated the feasibility of PET in the diagnosis of pleural thickening on CT. They concluded that qualitative assessment with PET discriminates between malignant and benign pleural thickening with high accuracy and a high negative predictive value. However, there has been no description of tuberculous pleurisy [15]. To the best of our knowledge, this is the first reported case of tuberculous pleurisy with intense FDP uptake.

The pathogenesis of primary tuberculous pleurisy is a delayed-type hypersensitivity immunogenic reaction to a few mycobacterial antigens entering the pleural space rather than direct tissue destruction by uncontrolled mycobacterial proliferation [12]. This appears to be the reason for the low rate of positive results in acid-fast staining of pleural fluid and tissue, as seen in this case [16]. Recent findings suggest that the immunogenic reaction in tuberculous pleurisy involves lymphocytes (including CD3, CD4, CCR4, and Th17 cells), neutrophils, mesothelial cells, and various cytokines such as interferon-γ, interleukin (IL)-8, IL-12, and monocyte chemoattractant protein-1 [17,18]. However, the complex mechanisms that induce high glucose metabolism and show high uptake of FDG in pleural thickening is unclear at this time.

About half of untreated cases of primary tuberculous pleurisy relapse with more severe forms of active pulmonary or extra-pulmonary tuberculosis, which may result in severe disability or death [19,20]. However, in general, many cases of pleural thickening suspiciously caused by primary tuberculous pleurisy with no subjective symptoms have not received antituberculous chemotherapy. FDG uptake in the pleura with positive QuantiFERON-TB may support the use of antituberculous treatment in such cases.

## Conclusions

Active tuberculosis should be considered in the differential diagnosis of diffuse pleural thickening even when there are no subjective symptoms. Moreover, FDG PET/CT imaging may be useful for evaluating disease activity and monitoring therapeutic responses in tuberculous pleurisy patients with an unknown time of onset.

### Consent

Written informed consent was obtained from the patient for publication of this case report and any accompanying images. A copy of the written consent is available for review by the Editor-in-Chief of this journal.

## Abbreviations

FDG: 18-fluorodeoxyglucose; PET: Positron emission tomography; CT: Computed tomography; SUVmax: Maximal standard uptake value; QFT-2G: QuantiFERON-TB second generation; IL: Interleukin.

## Competing interests

The authors declare that they have no competing interests.

## Authors’ contributions

TS was directly involved in the diagnosis and treatment of the patient, manuscript preparation, editing, and submission. NS contributed to patient care and collected patient data. MK performed the surgical procedure. KN performed pathologic studies. NH was the reference radiologist during the management of this case. FO was the attending physician throughout the disease course. All authors read and approved the final manuscript.

## Pre-publication history

The pre-publication history for this paper can be accessed here:

http://www.biomedcentral.com/1471-2334/13/12/prepub
